# An exploration of gut microbiota mechanisms underlying differential efficacy of vitamin D supplementation in children associated with lipid metabolism

**DOI:** 10.3389/fnut.2025.1670065

**Published:** 2025-11-13

**Authors:** Hui Wang, Wan-ying Liu, Tong-tong Wang, Ying-tan Yu, Xin-xu Li, Fei-tong Zhang, Chen-yang Zhang, Xiao Tong

**Affiliations:** 1 Affiliated Hospital of Jiangnan University, Wuxi, China; 2 Wuxi Medical College, Jiangnan University, Wuxi, China

**Keywords:** vitamin D, children’s gut microbiota, lipid metabolism, 16S rRNA, PICRUSt2

## Abstract

**Introduction:**

Vitamin D plays an indispensable role in children’s growth. Although there has been a growing recognition of the importance of vitamin D supplementation, its deficiency remains a prevalent global issue. This deficiency is frequently associated with abnormalities in lipid metabolism, which can impede the normal development of children. In recent years, the gut microbiota, which exerts a significant influence on drug metabolism and nutrient absorption, has captured increasing attention. This study explores gut microbiota mechanisms underlying the differential efficacy of vitamin D supplementation related to lipid metabolism, offering references for clinical problem-solving.

**Methods:**

A retrospective analysis of 2,307 pediatric patients examined serum vitamin D levels and their correlation with lipid metabolism. To explore the potential mechanism, children with vitamin D deficiency underwent a 3-month supplementation and 1 h of outdoor activities each day. Then, 24 subjects were selected (12 poor and 12 good responders to supplementation). 16S rRNA sequencing was used to analyze gut microbiota composition to find differential microbiota, and PICRUSt2 was used for functional prediction to identify potential metabolic differences.

**Results:**

Our study revealed a widespread prevalence of vitamin D insufficiency or deficiency among children. Moreover, a negative correlation was established between vitamin D levels and lipid metabolism. The results of 16S rRNA sequencing indicated that *Agathobacter* emerged as the key gut microbiota influencing the efficacy of vitamin D supplementation. Its abundance was negatively correlated with lipid metabolism. Through PICRUSt2 analysis, distinct ‘other carbohydrate degradation’ pathways were identified in children with different responses to vitamin D supplementation.

**Discussion:**

This study confirms the widespread occurrence of vitamin D insufficiency or deficiency in children, which is negatively correlated with lipid metabolism. *Agathobacter* may influence vitamin D absorption and lipid metabolism through the ‘other carbohydrate degradation’ pathway. Our findings offer more insights for clinical problem-solving and new therapies based on child–gut microbiota interactions.

## 1 Introduction

Vitamin D plays a crucial and irreplaceable role in children’s growth and development. Traditionally, the focus has often been on its function in bone development. However, a growing body of research indicates that vitamin D receptors (VDRs) are widely distributed in various cell types, including immune cells, neurons, and pancreatic β-cells. This widespread distribution pattern suggests that vitamin D has significant potential to regulate multiple systems (1). Nevertheless, vitamin D deficiency in children is prevalent worldwide (2), with an incidence rate ranging from 30 to 80% (3). In China, the proportion of vitamin D deficiency and insufficiency among healthy children and adolescents (aged 0–18 years) is approximately 28.71%, and the proportion of insufficiency gradually increases with age (4). Although oral vitamin D supplements are widely used in clinical practice, individual differences and underlying conditions such as obesity may reduce their effectiveness (2). Research has shown that there is a bidirectional relationship between abnormal lipid metabolism and vitamin D deficiency, and the underlying mechanisms are extremely complex. Vitamin D metabolic disorder is one of the key factors and is regarded as a core regulatory hub (5). Vitamin D, in its active form 1,25-dihydroxyvitamin D3, binds to vitamin D receptors (VDRs) and regulates gene expression through multiple pathways, including adipocyte differentiation, lipid storage, inflammation, and oxidative stress, thereby influencing lipid metabolism (6, 7). Previous animal experiments conducted by our team have also confirmed that vitamin D deficiency can lead to lipid metabolism disorders in mice through the triglyceride metabolism pathway (8). Serum 25(OH)D levels in obese individuals are generally low, which may be closely related to the ‘sequestration effect’ of adipose tissue on vitamin D (9, 10). In recent years, the gut microbiota, as a key factor affecting drug metabolism and nutrient absorption, has attracted increasing attention. This ecosystem has dual functions and heterogeneity and is often vividly compared to a ‘metabolic organ’ (11, 12). Compared with the relatively stable human genome, the gut microbiota exhibits remarkable adaptability and fluidity (13, 14). It can not only directly affect drug metabolism and toxicity (15) but also play an important role in human nutrient intake, immune regulation, and the occurrence and development of diseases (14, 16, 17). Relevant literature shows that there are mutually influencing pathways between the gut microbiota, lipid metabolism, and vitamin D levels. For example, probiotics such as *Lactobacillus reuteri* can promote lipid emulsification by regulating bile acid metabolism (18), thereby increasing the absorption rate of vitamin D. The latest research has also found that *Akkermansia muciniphila* can significantly reduce the body weight of obese mice by upregulating the secretion of glucagon-like peptide-1 (GLP-1) and improving intestinal barrier function (19). Clinical trials have also confirmed that the application of probiotics can regulate the gut microbiota structure in adolescent obese patients (20).

Given that the ‘sequestration effect’ of adipose tissue in individuals with abnormal lipid metabolism limits the effectiveness of vitamin D supplementation, the strategy of optimizing vitamin D supplementation and improving lipid metabolism by regulating the gut microbiota is a promising strategy and is expected to enhance the efficiency and safety of clinical drug use at the individual level.

## 2 Materials and methods

### 2.1 Study design

This study used a two-stage design. First, we analyzed the association between vitamin D levels and lipid metabolism in a large retrospective cohort to establish a macro-phenotypic correlation. A retrospective analysis was conducted using the database of the Paediatric Growth and Development Expert Clinic at Jiangnan University Affiliated Hospital. Serum 25-hydroxyvitamin D [25(OH)D] levels and blood biochemical indicators, including triglycerides (TG), total cholesterol (TC), and alkaline phosphatase (ALP) levels, were assessed in children who visited the clinic between 2021 and 2024. Building upon this foundation, to further investigate the underlying mechanisms behind this association, we conducted a second phase of exploratory analysis. This involved supplementing vitamin D in children with vitamin D deficiency and comparing the gut microbiota of individuals who responded markedly differently to supplementation. All medical records were anonymized. The study was approved by the Research Ethics Committee of Jiangnan University Affiliated Hospital (approval number LS2020046). All fecal samples were collected starting at 8:00 a.m. Participants were asked to avoid consuming substances containing caffeine or alcohol within 24 h prior to sample collection and to avoid antibiotic treatment within 3 months prior to collection. All participants were fully informed about the study and provided written consent. We confirm that informed consent was obtained from all participants and/or their legal guardians. We confirm that all methods were conducted in accordance with the relevant guidelines and regulations.

### 2.2 Inclusion and exclusion criteria

The inclusion criteria included: (1) patients who visited the Pediatric Growth and Development Expert Clinic at the Affiliated Hospital of Jiangnan University, (2) age between 6 and 15 years, and (3) daily supplementation of vitamin D as prescribed by a physician and 1 h of outdoor exercise within the past 3 months, with records of follow-up visits at the clinic.

The exclusion criteria included: (1) regular use of non-steroidal anti-inflammatory drugs (NSAIDs); (2) use of antibiotics within 3 months prior to the study or during the study period; (3) intestinal inflammation–inflammatory bowel diseases (including ulcerative colitis and Crohn’s disease, etc.); (4) gastrointestinal diseases; (5) allergic, genetic, chronic, or autoimmune diseases; and (6) regular consumption of probiotics, probiotic foods, or supplements prior to and/or during the study.

### 2.3 Participants

Between 2021 and 2024, 24 children aged 6–15 years with vitamin D deficiency and meeting the inclusion criteria were recruited from the Paediatric Growth and Development Expert Clinic at the Affiliated Hospital of Jiangnan University. The subjects were separated into two groups, with six males and six females in each group. Twelve cases whose 25(OH)D levels remained below normal after 3 months of vitamin D supplementation and 1 h of outdoor activity daily were assigned to the vitamin D deficiency (VDD) group. The remaining 12 cases, whose 25(OH)D levels significantly improved to normal levels after the same treatment, were assigned to the control (CON) group. The two groups were comparable at baseline.

### 2.4 Anthropometry and body composition assessment

All anthropometric measurements and bone density assessments were performed by the same trained and experienced nurse to ensure consistency of results. Measurement Standards: (1) Height: measured using a calibrated stadiometer, accurate to 0.1 cm. (2) Weight: measured using calibrated electronic scales, with participants wearing light clothing, accurate to 0.1 kg. (3) Body Mass Index (BMI): calculated as weight (kg)/height (m2). (4) Bone Mineral Density (BMD) levels details: whole-body fat mass, lean body mass, and bone mineral content were assessed via dual-energy X-ray absorptiometry (DXA) scanning (Equipment model: Sunlight MiniOmni, BeamMed Ltd., Petah Tikva, Israel). All scanning and analyses followed the manufacturer’s standard operating procedures. Daily quality control of the equipment was performed using manufacturer-provided standard phantoms.

### 2.5 Evaluation and analysis

#### 2.5.1 Assessment of vitamin D supplement compliance

We monitored participants’ vitamin D supplementation through regular structured telephone interviews. This included, but was not limited to, participants’ pill counts and medication diaries completed by pediatric patients and their families to monitor and evaluate supplement adherence. No significant differences in compliance were observed between the CON group and the VDD group.

① Vitamin D deficiency: serum 25(OH)D level <20 ng/mL, ② vitamin D insufficiency: serum 25(OH)D level 21–29 ng/mL, and ③ vitamin D sufficiency: serum 25(OH)D level ≥30 ng/mL.

#### 2.5.2 Statistical analysis

The data were analyzed using SPSS 27.0 software. Count data were analyzed using the chi-squared test and presented as case numbers (*n*) and percentages (%). A *t*-test was used to compare the two groups. Spearman’s correlation analysis was used to describe the relationship between TG, TC, ALP, and 25(OH)D levels. Then, skewed data were log-transformed to approximate a normal distribution, and a linear regression model was used to further assess the association between serum 25(OH)D levels and the above indicators.

#### 2.5.3 Analysis of serum levels of 25(OH)D and lipid markers in children

Formula: the formula for calculating Spearman’s rank correlation coefficient is:


$$\rho =1-\frac{6\sum_{i=1}^{n}d_{i}^{2}}{n(n^{2}-1)}$$

where *ρ* is the Spearman’s correlation coefficient, *d*_*i* is the difference in rank between two variables, and *n* is the number of observations. Value range: the value of Pearson’s correlation coefficient ranges from −1 to 1. A correlation coefficient of 1 indicates a perfect positive correlation, a correlation coefficient of −1 indicates a perfect negative correlation, and a correlation coefficient of 0 indicates that there is no linear relationship between the two variables.

### 2.6 Clinical fecal sample 16S rRNA analysis

#### 2.6.1 Fecal sample collection

Participants collected fecal samples using the containers provided by the Jiangnan University Affiliated Hospital. The samples were stored at −80 °C within 2 h of collection.

#### 2.6.2 16S rRNA sequencing and statistical analysis

Fecal samples were sent to an external company (APExBIO Technology LLC) to amplify the V3-V4 hypervariable region of the 16S rRNA gene. This was conducted using the Illumina NovaSeq 6000 in PE250 mode with the following primers: forward primer CCTAYGGGRBGCASCAG and reverse primer GGACTACNNGGGTATCTAAT. Raw data were obtained using this method. The Qiime 2 analysis workflow was then used, with DADA2 being applied to denoise the raw data by effectively clustering DNA sequences at 100% similarity. Low-quality sequences were removed and corrected, and the algorithm identified and removed chimeras. The denoised sequences were deduplicated directly to obtain feature information (these features are collectively referred to as OTUs, ASVs, etc.). To further explore differences in community structure between grouped samples, statistical analysis methods such as Adonis and Anosim were used to test the significance of differences in species composition and community structure. LEfSe analysis and Kruskal–Wallis (KW) tests were used for differential feature screening. Taxonomic analysis of the feature sequences was performed using the Naive Bayes classifier and the QIIME2 feature classifier, and the community composition of each sample was statistically analyzed at each taxonomic level.

#### 2.6.3 Alpha and beta diversity analyses

Alpha diversity is used to analyze the diversity of microbial communities within a sample. Single-sample diversity analysis (alpha diversity) reflects the richness and diversity of the microbial community within the sample. The Wilcoxon rank-sum test is used to analyze differences in alpha diversity indices between two groups, while the Kruskal–Wallis test is used for differences between multiple groups. Statistical distance analysis can quantify the extent of differences in species abundance distribution between groups. Four distance analysis methods were designed for this analysis, which were calculated using the Bray–Curtis, Jaccard, Unweighted_unifrac, and Weighted_unifrac algorithms to obtain distance matrices. These distance matrices were then used for beta diversity analysis and visualization. The *p*-value threshold for *α* and *β* diversity analysis was set at ≤0.05.

#### 2.6.4 Differential abundance analysis

The Wilcoxon rank-sum test (for 2 groups) was performed using R software to identify differentially abundant bacteria between groups. These bacteria were then sorted in descending order based on their relative abundance. LEfSe analysis, also known as LDA effect size analysis, enables comparisons between two or more groups and identifies species with significant differences in abundance (i.e., biomarkers). First, the non-parametric Kruskal–Wallis rank-sum test detects species with significant differences in abundance between groups. Then, the Wilcoxon rank-sum test determines whether differences in the previously identified species are consistent across groups. Finally, linear discriminant analysis (LDA) is used to estimate the effect of each species’ abundance on the difference. The default threshold for the log LDA value is 2; if it is greater than 2, the bacteria are considered to be significantly different between groups. When the *p*-value is less than 0.05, this indicates that the difference is statistically significant.

### 2.7 PICRUSt2 function prediction

PICRUSt2 can predict the relative abundance of microbial functional categories in a sample using 16S rRNA gene sequencing data. First, the software infers the gene functional spectrum of the common ancestor using full-length 16S rRNA gene sequences from the sequenced microbial genomes. It then constructs a gene functional prediction spectrum for the entire archaeal and bacterial domains. Finally, it “maps” the sequenced microbial community composition to the database to predict the functional composition of the microbial community.

## 3 Results

### 3.1 Vitamin D deficiency is prevalent among children, and there is a significant negative correlation between its levels and triglyceride levels

**Figure 1:**
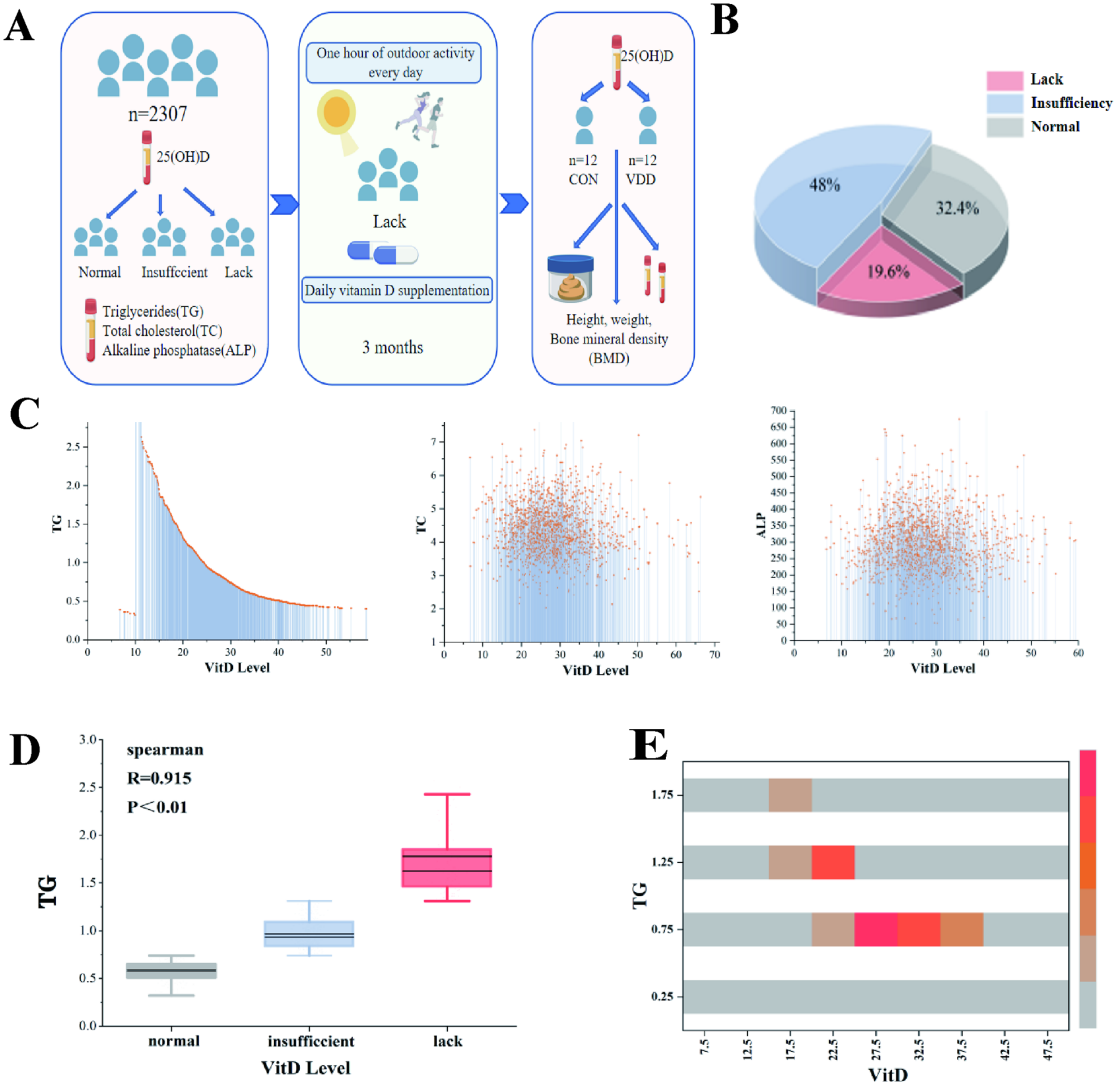
Overall research design and the correlation between vitamin D levels and lipid metabolism levels in children. **(A)** Experimental design flowchart. **(B)** Among outpatient children, 19.6, 48, and 32.4% had vitamin D deficiency, insufficiency, and normal levels, respectively. **(C)** A linear relationship was observed between vitamin D levels and TG, TC, and ALP levels. **(D)** Spearman’s correlation analysis between vitamin D levels and TG (*R* = 0.915, *p* < 0.01). **(E)** The heatmap shows the correlation analysis between TG and vitamin D levels.

In order to investigate 25(OH)D levels in children and their correlation with lipid metabolism, 2,307 original case records were collected and organized from the Paediatric Growth and Development Expert Clinic at the Affiliated Hospital of Jiangnan University (Table 1). Analysis of the aforementioned children’s serum 25(OH)D levels revealed that 19.6% (448/2307) and 48% (1096/2307) had vitamin D deficiency and insufficiency, respectively, while only 32.4% had normal levels. This suggests that vitamin D levels are generally inadequate in this pediatric population (Figures 1A,B).

**Table 1:** Summary table of 25(OH)D levels in pediatric patients at the Children’s Growth and Development Specialist Clinic of Jiangnan University Affiliated Hospital (2021–2024).

Vitamin D levels	Number of cases (*n*)	Percentage (%)	25(OH)D (ng/mL)
Normal	740	32.4%	≥30
Insufficient	1,096	48%	20 ≤ *X* < 30
Lack	448	19.6%	<20
Total	2,307	100%	

We conducted correlation analyses between 25(OH)D levels and biochemical indicators of lipid components, including TG, TC, and ALP. The results showed a significant linear relationship between 25(OH)D levels and TG (Figure 1C). Further analysis using Spearman’s correlation coefficient revealed a significant negative correlation between serum 25(OH)D levels and TG (*p* ≤ 0.01), with no such relationship observed for TC or ALP (Figure 1D). A correlation analysis heatmap also visually confirmed this result (Figure 1E).

These results show that a significant proportion of children in this pediatric population do not have normal 25(OH)D levels and that there is a significant negative correlation between serum 25(OH)D levels and TG levels.

### 3.2 Equation analysis of the gut microbiota of the two groups of children in a case-control study

#### 3.2.1 Overall gut microbiota composition

**Figure 2:**
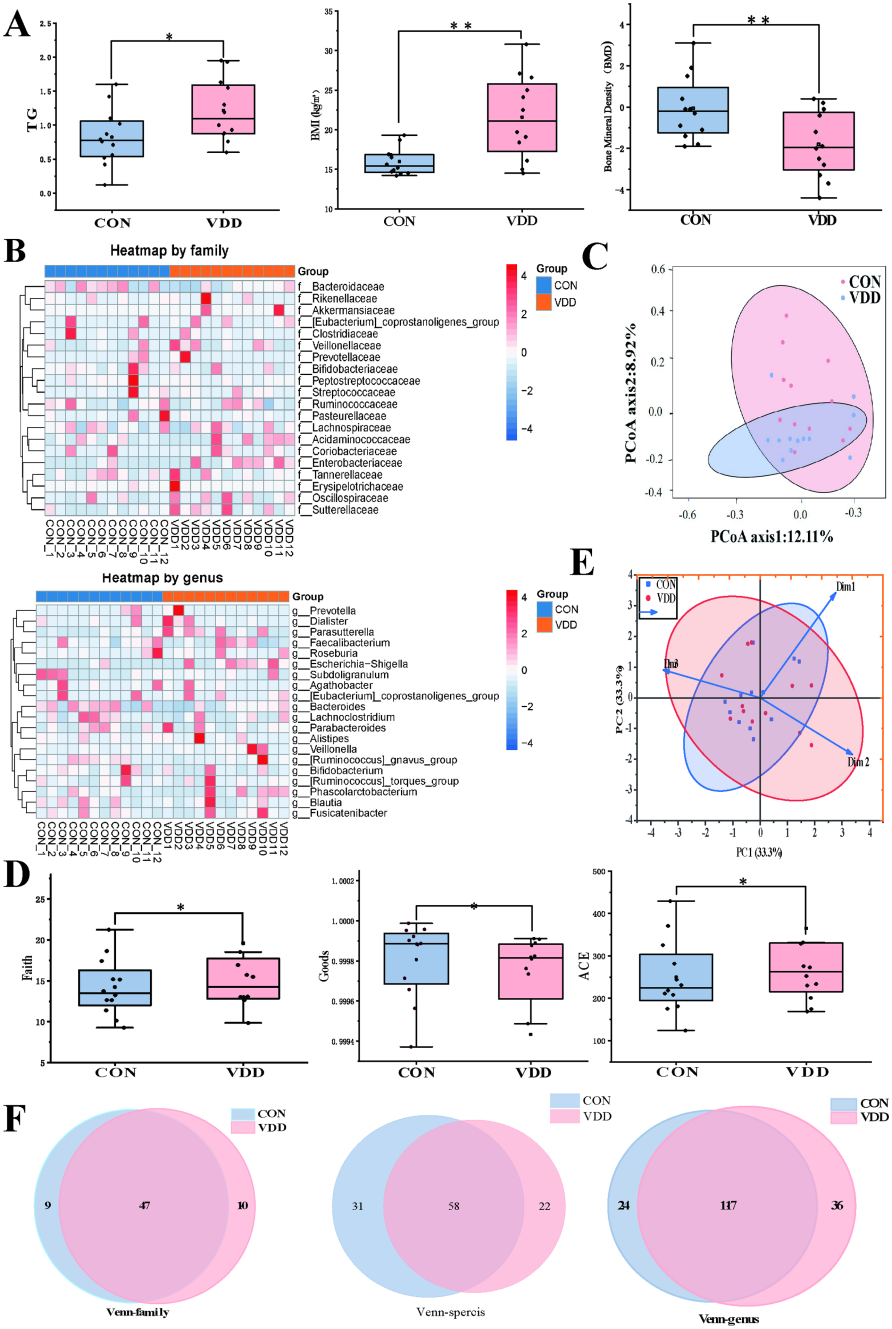
Comparison of the differences in lipid metabolism levels and gut microbiota abundance between the two groups. **(A)** The VDD group exhibits lower levels of lipid metabolism and bone mineral density (BMD) compared to the CON group. (TG: *p < 0.05, BMI, BMD: **p < 0.01). **(B)** The top 20 heatmap analyses show the abundance of intestinal microbiota at the family and genus levels. The redder the color, the higher the abundance of microbiota; the bluer the color, the lower the abundance. **(C)** Principal coordinates analysis (PCoA), where the distance between sample points represents the similarity of microbial communities between the two groups: the closer the distance, the greater the similarity. **(D)** Comparison of the faith index, the ACE index, and the goods index of gut microbiota between the two groups. **(E)** Beta diversity PCoA plot shows the degree of difference in species abundance distribution between groups. **(F)** Analysis of shared and unique amplicon sequence variants (ASVs) across different groups, with Venn diagrams plotted at family, genus, and species levels.

We analyzed the clinical indicator data measured in the two groups and performed 16S rRNA analysis of the gut microbiota in the collected fecal samples. The results showed that the TG levels and body mass index (BMI) of the CON group were significantly lower than those of the VDD group, and that their bone mineral density levels were significantly higher (Figure 2A). There were no statistically significant differences in the basic information of the subjects (Table 2). Next, we performed 16S rRNA analysis on the fecal samples. Analysis of a heatmap showing the top 20 families and genera of gut microbiota revealed that the most abundant phylum among the identified bacterial communities was *Firmicutes*, followed by *Bacteroidetes* (Figure 2B). Principal coordinate analysis (PCoA) results showed differences in microbial communities between the CON group and VDD (Figure 2C).

**Table 2:** Clinical characteristics of pediatric patients enrolled in two groups prior to enrollment.

Clinical characteristics	CON (12 cases)	VDD (12 cases)	*p*-value
Age (years)	10.0	11.0	0.31
Female/male (cases)	6/6	6/6	1.00
25(OH)D (ng/mL)	17.94 ± 0.176	17.96 ± 0.193	0.84
Height (cm)	141.1 ± 13.77	152.08 ± 13.62	0.06

We performed *α* and *β* diversity analyses on two groups of fecal samples. The *α* diversity analysis revealed differences in species richness between the VDD and CON groups (Figure 2D). The PCoA plot showed the *β*-diversity of the two groups, indicating that most of the core microbiota shared by the two groups constituted the main body of the community and were similar. However, differences exist among certain non-core, low-abundance, or highly plastic bacterial genera (Figure 2E). A comprehensive review of the gut microbiota status of children in the VDD group revealed an upward trend in gut microbiota abundance at the family and genus levels, while abundance decreased at the species level (Figure 2F).

#### 3.2.2 Differential abundance analysis

**Figure 3:**
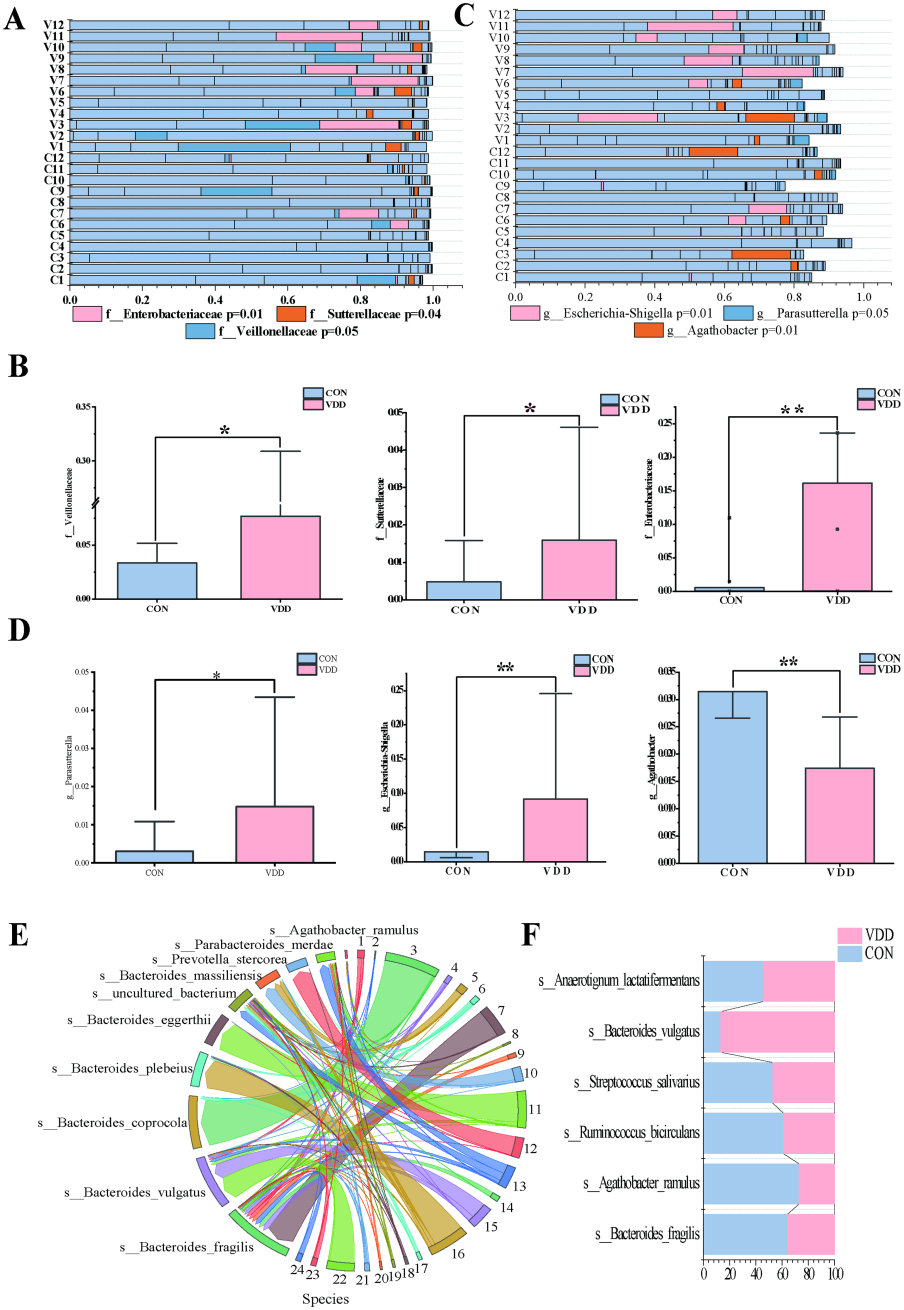
Difference in the abundance of *Agathobacter* between the two groups. **(A,B)** Analysis of differentially abundant bacteria between the two groups at the family level (*p < 0.05, **p < 0.01, ***p < 0.001). **(C,D)** Analysis of differential bacteria between the two groups at the genus level (*p < 0.05, **p < 0.01, ***p < 0.001). **(E,F)** String diagram of gut microbiota species distribution, showing the differences in species with higher abundance between the two groups.

Next, we analyzed the differential microbiota at different levels to investigate these differences in more detail. Further analysis revealed that the abundance of certain specific taxonomic groups was generally elevated in the VDD group. Specifically, the *Veillonellaceae* family, belonging to the *Firmicutes* phylum, and the *Enterobacteriaceae* family and *Sutterellaceae* family, belonging to the *Proteobacteria* phylum, were particularly prominent in this trend (Figures 3A,B). It is worth noting that *Proteobacteria* account for approximately 15% of the gut microbiota, and this phylum includes some potentially pathogenic bacteria, whose increase in proportion is associated with dysbiosis of the gut microbiota. Based on the above research findings, we can infer the following: although no obvious intestinal inflammation or other gastrointestinal diseases were observed in the selected population, those with poor vitamin D supplementation still had dysbiotic intestinal microbiota that could potentially trigger inflammatory changes. Furthermore, this abnormal state of the microbiota would adversely affect the absorption of nutrients such as vitamin D. *Parasutterella* and *Escherichia-Shigella* at the genus level were significantly more abundant in the VDD group than in the CON group (Figures 3C,D). These inferences can reasonably explain this phenomenon.

It is worth noting that the abundance of the *Agathobacter* genus within the *Lachnospiraceae* family was significantly higher in the CON group than in the VDD group (Figures 3C,D). Further analysis of the *Agathobacter* genus revealed that the abundance of *Agathobacter* ramulus (formerly known as *Eubacterium ramulus* and *A. ramulus*) in the intestinal microbiota of the CON group was higher than that in the VDD group (Figures 3E,F).

**Figure 4:**
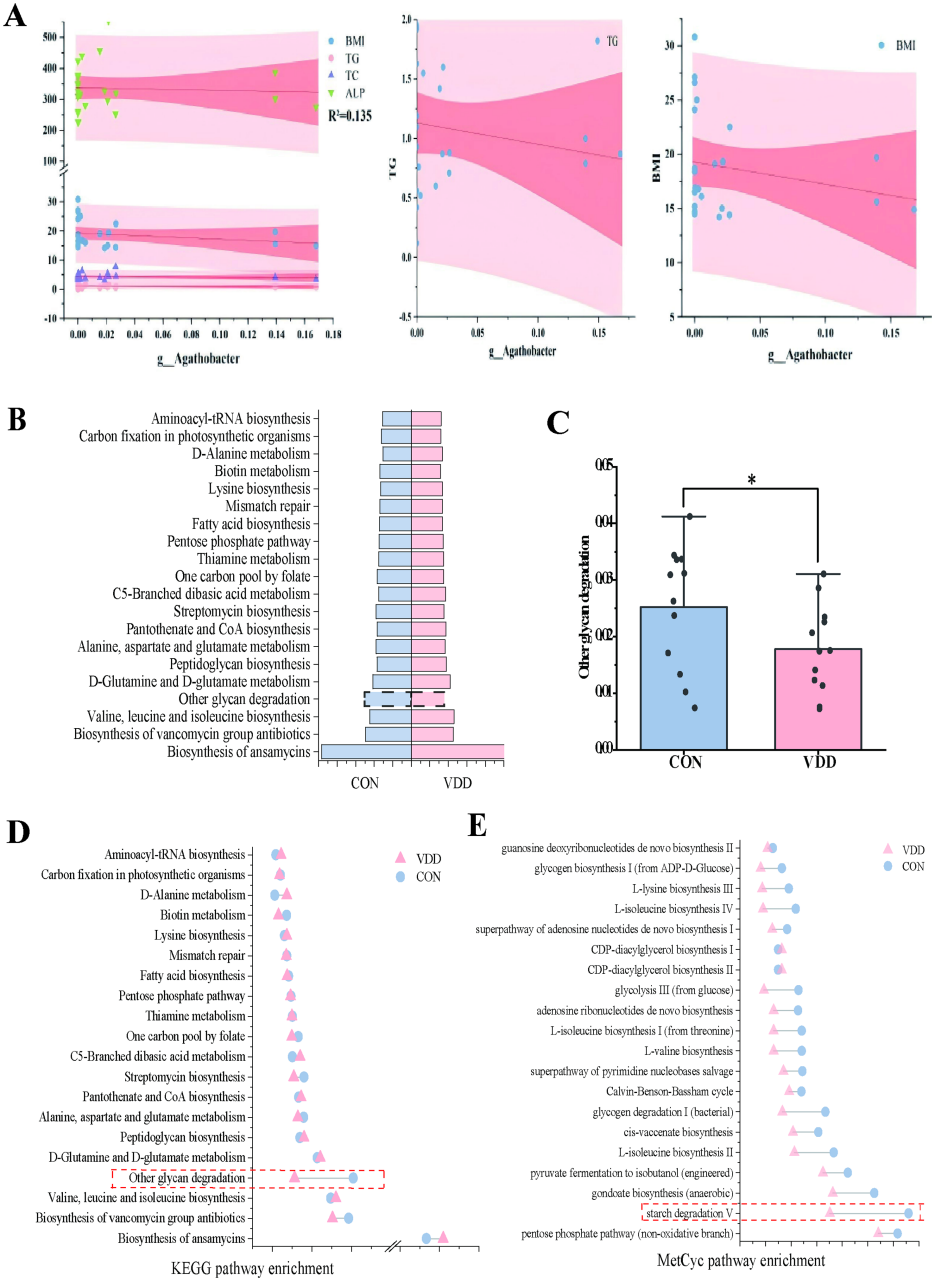
Differential analysis of metabolic pathways. **(A)** Multivariate linear regression analysis of the differences between bacteria *Agathobacter* and clinical measurement data (*R*^2^ = 0.135). **(B)** Heatmap analysis of the top 12 selected pathways in the CON and VDD groups, combined with the KEGG database. **(C)** A population pyramid shows the top 20 KEGG pathways enriched based on differential metabolism. **(D)** Differences between the two groups in the ‘Other Glycoside Degradation’ pathway (*p < 0.05). **(D,E)** Lollipop plots combine the KEGG and MetCyc databases describing differentially enriched metabolic pathways.

What effect does *Agathobacter* have on lipid metabolism in children? We conducted a linear regression analysis of *Agathobacter* alongside body mass index (BMI) and multiple biochemical indicators, using 24 clinical samples. While the explanatory power of the model was limited (*R*^2^ = 0.135) due to the influence of complex factors, it suggested that BMI and biochemical indicators such as triglycerides, total cholesterol, and alkaline phosphatase can still affect changes in *Agathobacter* abundance, with a negative correlation trend observed between Agathobacter and these indicators (Figure 4A). Based on these results, we can reasonably infer that, if the analysis is expanded to include a large number of clinical samples, these variables will exhibit a significant negative correlation.

### 3.3 Functional prediction

PICRUSt2 can ingeniously map the information on the microbial community composition obtained from 16S rRNA gene sequencing to multiple databases, thereby achieving an accurate prediction of the functional composition of the microbial community. In order to ascertain the functional disparities between the two groups, we conducted an enrichment analysis utilizing the KEGG and MetaCyc databases. The functional enrichment heatmap revealed these differences (Figure 4B). Metabolic pathway enrichment analysis using the KEGG database revealed that activity in the ‘other glycoside degradation’ pathway was higher in the CON group than in the VDD group (Figures 4C,D). In addition to the differences observed in the ‘other glycoside degradation ‘pathway, analysis using the MetaCyc database revealed that the ‘starch degradation V’ pathway exhibited greater activity in the CON group than in the VDD group (Figure 4E).

In summary, we found that the CON group exhibited higher levels of vitamin D absorption and utilization, as well as improved lipid metabolism. This may be due to the differential bacterium *Agathobacter* affecting the pathways involved in ‘Other glycan degradation’ and ‘starch degradation V’.

## 4 Discussion

In recent years, issues such as obesity, vitamin D deficiency, and insufficiency among children have received significant attention, leading to an increasing number of studies aimed at addressing these problems. The gut microbiota plays a crucial role in nutrient absorption and utilization, so we have focused on the microbiological mechanisms underlying these issues in children and explored the metabolic pathways through which they exert their effects. In clinical practice, if the goal is to enhance vitamin D absorption and utilization by modifying the intestinal microenvironment, given the broad-spectrum antibacterial properties of current antibiotics, it is challenging to precisely target and eliminate a specific bacterial group. Therefore, we may consider identifying and supplementing with potentially beneficial bacteria that may be present.

We conducted a clinical retrospective analysis, 16S rRNA analysis of the gut microbiota in two groups of pediatric patients, and functional enrichment analysis. By analyzing 2,307 original samples from the growth and development clinic, we found an association between serum 25(OH)D levels in children and lipid metabolism (particularly triglyceride levels). 16S rRNA gut microbiota analysis identified *Agathobacter* as a potential beneficial bacterial species promoting vitamin D absorption and lipid metabolism. Existing research has shown that *Bifidobacteriaceae* and *Christensenellaceae* are closely associated with vitamin D levels (21). *Agathobacter* is a core member of the Firmicutes phylum that primarily colonizes the human colon. It produces butyrate through the fermentation of dietary fiber, such as pectin and resistant starch (22). Butyrate, which enhances hepatic fatty acid oxidation by activating the AMPK pathway. This reduces lipid accumulation and promotes vitamin D absorption and transport (22, 23). Furthermore, butyrate promotes the differentiation of regulatory T cells (Tregs) and inhibits the progression of ulcerative colitis (24). Studies have shown that butyrate, produced by *Agathobacter,* activates the peroxisome proliferator-activated receptor gamma (PPARγ) pathway in intestinal epithelial cells, upregulates zonula occludens 1 (ZO-1), reduces intestinal permeability (thereby reducing ‘leaky gut’), and improves the efficiency of trans-epithelial absorption of vitamin D (22). G protein-coupled receptors (GPCRs) are key mediators of butyrate’s anti-inflammatory effects. Butyrate can inhibit intestinal inflammation by activating the GPR109A receptor, thereby promoting the repair of the colonic epithelial barrier (25). As a member of the *Agathobacter* genus, *A. ramulus* plays a crucial role in metabolic regulation, immune function maintenance, and neuroprotection, and holds potential as a target for disease intervention.

Further functional enrichment analysis explored potential metabolic mechanisms, specifically the ‘other carbohydrate degradation’ and ‘starch degradation V’ metabolic pathways. ‘Other glycan degradation’ refers to the breakdown of polysaccharides such as cellulose, hemicellulose, and pectin, which are found in dietary fiber. This process is primarily carried out by gut microbiota, which break these substances down into short-chain fatty acids (SCFAs), including acetate, propionate, and butyrate (26). Starch degradation V specifically refers to the metabolic pathway by which gut microbiota break down V-type-resistant starch (RS5). RS5 is a single-helix complex formed by hydrophobic interactions between linear starch and ligands such as lipids, polyphenols, or proteins. Therefore, compared to the degradation of conventional RS2/RS3 starch, RS5 exhibits high resistance to enzymatic hydrolysis. After degradation by microbiota, over 60% of the resulting products are butyrate, followed by propionate (27).

Our findings suggest that *Agathobacter* may promote vitamin D absorption and lipid metabolism disorders by influencing the aforementioned metabolic pathways.

Based on the above research, we can conclude that the gut microbiota *Agathobacter* may play a key role in children’s serum 25(OH) levels and lipid metabolism by influencing the metabolic pathways ‘other carbohydrate degradation’ and ‘starch degradation V’, thereby affecting their growth and development.

We will further elucidate the roles and mechanisms of specific bacterial strains in health and disease, to improve lipid metabolism disorders, enhance vitamin D absorption and utilization, and address obesity and related health issues in children with growth and development disorders through the use of probiotics and their derivatives.

This study has several advantages. First, the study population is more precisely defined, focusing on children at the growth and development stage. Second, the clinical observational data are more accurate, covering indicators related to vitamin D levels and lipid metabolism. Third, clear inclusion and exclusion criteria were established to ensure that participants with certain diseases or conditions, or who were taking medications that directly affect the gut microbiota, were not included in the study. This makes the correlation between the efficacy of vitamin D oral therapy, lipid metabolism, and the gut microbiota in the pediatric population clearer. However, this study also has certain limitations. First, 16S rRNA sequencing technology was used. Although this is one of the most commonly used techniques for analyzing the gut microbiota, it has drawbacks such as low resolution and an inability to provide functional analysis. It is also subject to inherent biases related to primer selection and targeting highly variable regions. Second, the study population placed greater emphasis on growth- and development-related indicators than the general population. Third, future research should involve larger sample sizes for validation. Fourth, assessing the gut microbiome using fecal samples has certain limitations.

The results of this study suggest that the gut microbiota of children can significantly impact the effectiveness of vitamin D supplementation and influence lipid metabolism. These findings could inform the development of future intervention strategies aimed at improving gut microbiota as a non-pharmacological approach to promoting children’s health. While this study examined the microbiome associated with differences in the efficacy of vitamin D supplementation in children with vitamin D deficiency related to lipid metabolism, the underlying mechanisms have yet to be fully validated. Future research should focus on clinical translation to address clinical issues through the development of probiotics and prebiotics.

## 5 Conclusion

This study investigated the microbiological mechanisms underlying the varying efficacy of vitamin D supplementation in children with vitamin D deficiency and its impact on lipid metabolism. The study revealed a widespread prevalence of vitamin D deficiency in the pediatric population and confirmed the negative association between vitamin D levels and lipid metabolism. It also identified the bacterium *Agathobacter* and its potential influence on metabolic pathways. These findings emphasize the importance of gut microbiota in children, highlighting the connection between gut microbiota, the effects of vitamin D supplementation, and lipid metabolism. This suggests that gut microbiota could be used to support children’s health and promote growth and development. Future research will use gut-derived metabolomics analysis to further understand the functional characteristics of the gut microbiota and its relationship with vitamin D supplementation and lipid metabolism. This will improve our understanding of the connections among the three factors. Future research should focus on translational medicine, utilizing gut microbiota and its derivatives as new biopharmaceuticals to improve children’s health.

## Data Availability

The raw data supporting the results of this study are not publicly available due to sensitivity reasons and can be obtained from the corresponding author upon reasonable request. The data are stored in controlled access data storage at the Affiliated Hospital of Jiangnan University.
